# Experimental and Numerical Analysis of 60-Year-Old Sluice Gate Affected by Long-Term Operation

**DOI:** 10.3390/ma13225201

**Published:** 2020-11-17

**Authors:** Miroslav Pástor, Jozef Bocko, Pavol Lengvarský, Peter Sivák, Patrik Šarga

**Affiliations:** 1 Department of Applied Mechanics and Mechanical Engineering, Faculty of Mechanical Engineering, Technical University of Košice, 042 00 Košice, Slovakia; miroslav.pastor@tuke.sk (M.P.); jozef.bocko@tuke.sk (J.B.); peter.sivak@tuke.sk (P.S.); 2 Department of Automation and Human Machine Interactions, Faculty of Mechanical Engineering, Technical University of Košice, 042 00 Košice, Slovakia; patrik.sarga@tuke.sk

**Keywords:** sluice gate, safe operation, stress and strain analysis, residual stresses

## Abstract

Technological units of water reservoirs and power plants include sluice gates which are designed to completely seal the inflow or outflow of water in supply or discharge channels. This article describes the issue of technical assessment of a sluice gate made in the 1950s. Such structures are characterised by states of significant corrosive wear, permanent deformations of contact and sealing surfaces as well as increased levels of residual stresses. In such cases, it is difficult to determine service life using only numerical modelling methods, mainly due to problematic definition of material properties and boundary conditions. Therefore, for safety assessment, it is necessary to verify these facts experimentally. This article presents the procedure for assessing safe operation of the sluice gate on which places with permanent deformation and a broken part of the guide wheel flange were identified. By means of numerical modelling, we identified critical stress values at the locations of reinforcing elements, which were modified, and the stress values were reduced by about 15%. The results of numerical modelling were verified at select locations by experimental measurements during operation using strain gauges. The maximum values of operational normal stresses in the assessed places reached about 27 MPa. Based on the comparison of obtained results and taking into account values of residual stresses reaching up to 190 MPa made by shielded metal arc welding, it can be stated that, for safe operation of the sluice gate, it is necessary to follow the proposed procedure during its lowering and to modify the reinforcing elements structurally.

## 1. Introduction

Sluice gates, which are used as an integrated part of technological units of water reservoirs and power plants, block (close) the inflow or outflow of water in supply or discharge channels. Their correct operation is conditioned by correct design, production as well as the maintenance of a flawless technical condition during the expected period of operation. Consequences of their failure can be associated with great material and environmental damage or even death of people. Given the above reasons and the long-term operation of these plates, it is necessary to place great emphasis on their regular inspection and maintenance as well as on the processes of continuous professional monitoring of their technical condition [1,2,3]. From a structural point of view, these plates are mostly welded steel structures. Many of them were designed and manufactured several decades ago in accordance with regulations and standards in force at that time. As these standards and regulations were applied, which from today’s point of view are conservative and limited procedures, such plates were in most cases oversized in terms of strength and stiffness. Paradoxically, this is the reason why many such structures have survived in a more or less functional state to the present day. The purpose of technical capability verification is to predict the residual service life as accurately as possible. This service life is determined by two groups of factors. The first group includes structural and technological factors, such as geometry (shape and dimensions of the structure), assembly of the structure, structural characteristics, machining of functional surfaces, condition and character of notches, welds and other joints [4,5,6,7,8,9,10]. The second group of factors consists of operating characteristics such as structural load and environment (corrosion, high or low temperatures, radiation, etc.) [11,12,13,14,15]. The human factor (operation of the equipment) is also important.

Therefore, the following calculation and experimental procedures are most frequently used to assess the technical capability of load-bearing structures after long-term operation:

- assessment of material loss due to corrosion;
- determination of material properties;
- assessment of mechanical properties of the construction material due to its degradation after long-term operation, especially in structures affected by temperature, i.e., mainly in structures made from materials with creeping tendency;
- computational verification of strength, deformations and stability of structures;
- experimental determination of deformation and strength properties of structures in operating conditions, evaluation and analysis of data indicating technical condition of the structure;
- experimental determination of residual stresses taking into account production technology of the structure and the effect of operational overload; and
- experimental-computational assessment of fatigue damage accumulation and estimation of residual life.

**Figure 1:**
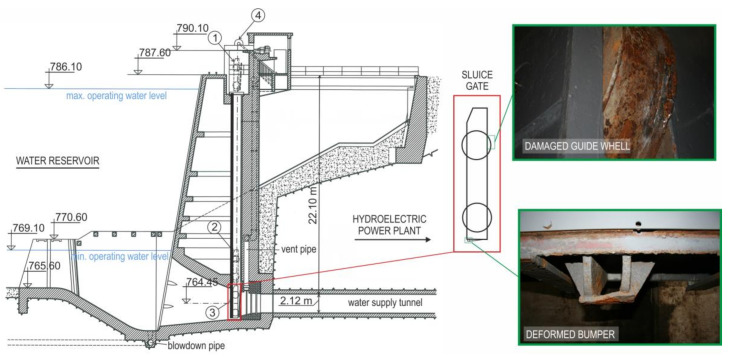
Section of the inlet to the supply tunnel with a deformed contact part of the sluice gate (bumper) and with a damaged part of the sluice gate (guide wheel).

The aim of this article is to assess safe operation of the 60-year-old sluice gate designed to block the inlet of the water tunnel connecting the water reservoir to the power plant (Figure 1) [16].

It is the first pumped storage power plant in Slovakia. It was built as a high-pressure plant in which two high-pressure Francis turbines and two generators with the total output 24 MW were installed. During planned maintenance, the deformed contact element of the plate, the so-called bumper (Figure 1, right top) and broken flange on one guide wheel (Figure 1, right bottom) were identified. The plate was located below the water surface for a long time just above the inlet of the blocked water tunnel (Figure 1, position 2) and, if necessary, it is ready to close the water tunnel immediately (Figure 1, position 3). In terms of its load, it can be stated that we are dealing with quasi-static load with hydrostatic water pressure in the tank. Faultless operation of the sluice gate and reliable blocking of the inlet are extremely important, especially in situations where there is technical staff in the water tunnel performing inspection and maintenance. The lifting and lowering mechanism of the sluice gate is located directly above the inlet to the supply tunnel (Figure 1, position 4).

**Figure 2:**
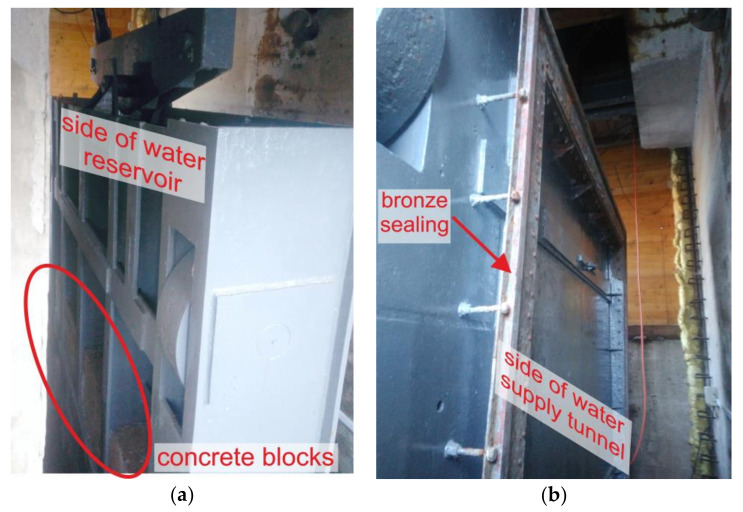
View of the sluice gate: (**a**) from the side of the water reservoir and (**b**) from the side of the water tunnel.

The sluice gate consists of a welded steel structure filled in the lower part with concrete blocks which serve as a load mass (Figure 2a). The ribs of the sluice gate consisting of I-profiles are connected by reinforcing elements. They are covered on the tunnel side with a steel plate to which bronze sealing bars are attached (Figure 2b).

The supporting structure of the sluice gate must be designed and manufactured in such a way that it can withstand, with an appropriate degree of reliability, loads which may occur during its operation, especially in lowering and lifting phases. With respect to the function of the sluice gate, its possible damage might cause not only great material damage but also a direct threat to human lives, which emphasizes the importance of verifying its safe operation. Experimental and numerical methods of mechanics were used to assess future safe operation.

## 2. Determination of Mechanical Properties

**Figure 3:**
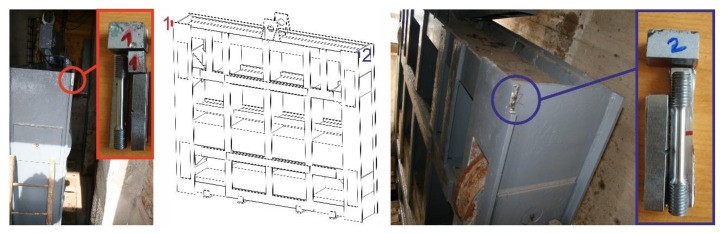
Locations of material samples.

In practice, there are cases when information is not available to perform relevant analyses regarding the material used, especially its chemical composition, material properties, etc. This is especially the case with older, earlier designed and manufactured structures. In such cases, material characteristics are most often determined on the basis of analyses of material samples. In our case, material samples were taken from two different locations (Figure 3). These locations were chosen to maintain integrity, functionality and strength of the sluice gate.

**Figure 4:**
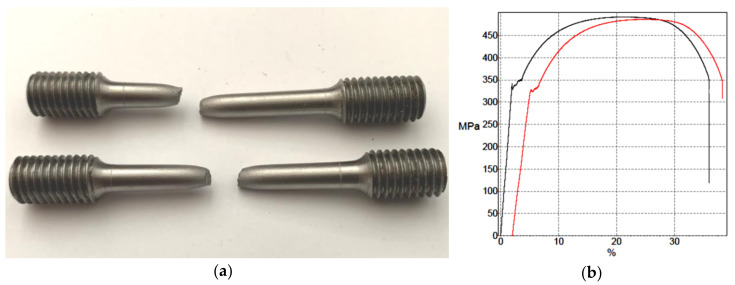
Tensile test: (**a**) test specimen and (**b**) tensile diagram.

Mechanical properties of the material were determined by a tensile test on a 200-kN Zwick extensometer (Ulm, Germany) on rods with circular cross section (Figure 4) according to the procedure prescribed in ISO 6892-1 [17]. The obtained material characteristics are given in Table 1.

**Figure 5:**
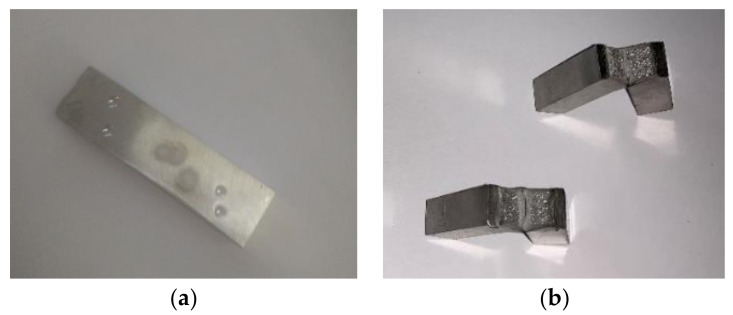
Test specimens: (**a**) for Brinell chemical composition and hardness measuring points and (**b**) for the impact test.

Material samples were then used to determine chemical composition, mechanical properties, hardness and impact strength and for purposes of a metallographic analysis. Chemical composition was determined by the spark method using a Belec Compact Port Spectrum Analyser (Belec Spektrometrie Opto-Elektronik GmbH, Georgsmarienhütte, Germany). The result of the measurements (Figure 5a) provides information on the mass ratio of individual chemical elements given in Table 2.

The Brinell hardness test was performed on the HPO 3000 apparatus according to the procedure specified in ISO 6506-1 [18] using a hemispherical penetrating body with a diameter of 10 mm made of carbide (HBW) (Figure 5b). The surface of test specimens was machined by grinding. The hardness values from the six measurements ranged from 132 to 137 HBW. The impact strength was measured within the impact test ISO 148-1 [19] with three-point bending of small test specimens with a sharp “V” notch (Figure 5b). The test was performed at room temperature. The dimensions of the test sample were 55 × 10 × 10 mm. Based on Figure 5b, the brittle fracture that would be unequivocally confirmed by SEM analysis can be observed. With regard to the obtained mechanical (strength) properties of steel, it can be stated that, for the given test conditions, the surface analysis is only supplementary. The values of impact performance (KC) and impact strength (KVC) are given in Table 3.

**Figure 6:**
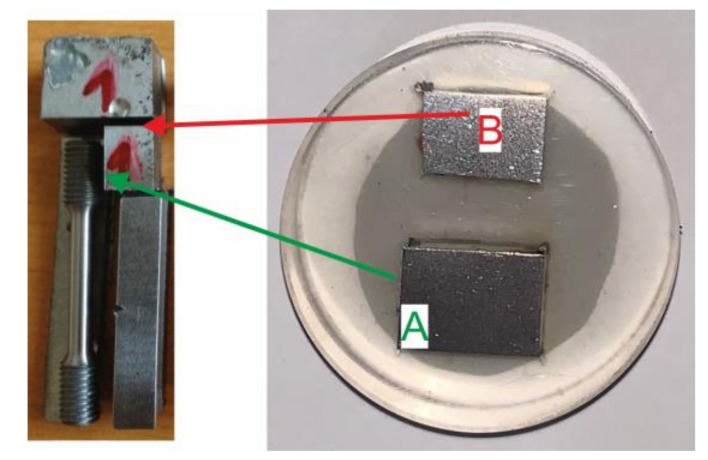
Metallographic cut in longitudinal direction (area A) and transverse direction (area B).

**Figure 7:**
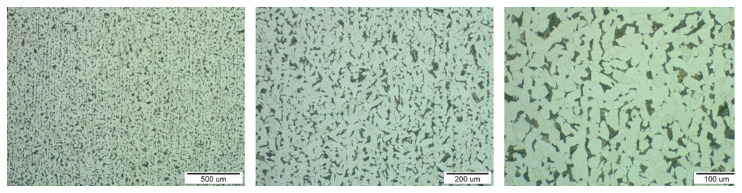
Sample microstructure in longitudinal direction (area A) at three different magnifications.

**Figure 8:**
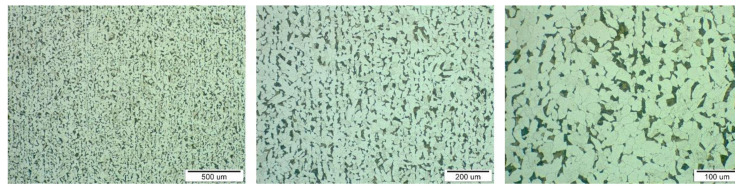
Sample microstructure in transverse direction (area B) at three different magnifications.

Metallographic structures of samples taken in longitudinal (Figure 6, area A) and transverse directions (Figure 6, area B) together with the respective scales are shown at different magnifications in Figure 7 and Figure 8. The analysis was performed on a light microscope Olympus VANOX–T (Tokyo, Japan). The structure can be characterised as ferritic-pearlitic with a low proportion of perlite. In the transverse direction, there is a structure with an indication of a row arrangement.

Based on the evaluation of performed tests and chemical analyses, it can be stated that the sluice gate material is a structural weldable low-carbon steel with a ferritic-pearlitic structure and that its properties correspond to steel 1.0040 [20] with yield strength R_e_ = 235 MPa and strength R_m_ = 412–510 MPa. It should be noted that the yield strength of the analysed material is significantly higher (approximately 330 MPa).

## 3. Determination of Residual Stresses on Sluice Gate

In practice, very little attention is paid to the issue of residual stresses, despite their frequent occurrence and their significant impact on reliability of the load-bearing elements of machines and equipment. Knowledge and consideration of residual stresses is extremely important, especially in welded structures in which they occur mainly due to significant temperature effects in the area around the weld and subsequent uneven cooling. Residual stresses in a structure can arise not only during its production but also during repairs or as a result of possible overload [21,22,23,24,25,26]. These stresses are superimposed on stresses from the operating load, which can reduce, sometimes substantially, safety and reliability of the load-bearing element and thus of the entire structure.

**Figure 9:**
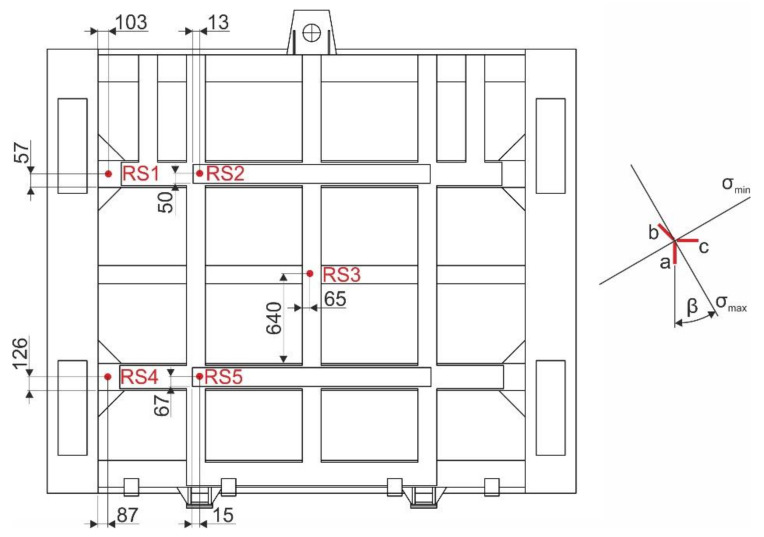
Position of strain gauge rosettes on the sluice gate.

For the measurement of residual stresses, a semi-destructive drilling method was chosen in accordance with ASTM E837-13a [27]. When choosing the measuring points, the authors relied on professional knowledge as well as their own experience gained when solving similar tasks of welded steel structures [28,29,30]. Figure 9 shows positions “RS1” to “RS5” where strain gauges were applied, including the orientation of individual grids. When selecting these measuring points, the current state of the steel structure of the sluice gate was also taken into account, especially the surface unevenness.

**Figure 10:**
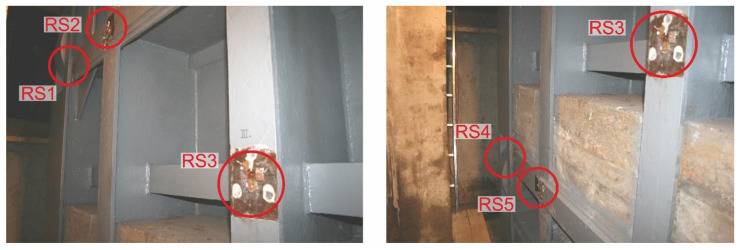
Position of strain gauge rosettes on the sluice gate.

Strain gauges from the HBM, type RY21-3/120, series A 412/01, identification number 8120 54419 with ohmic resistance of 120 Ω and a k-factor 2.06 on all grids were used to measure the residual stresses. Strain gauges were applied with Z70 adhesive and insulated with SG-250 and ABM-75 preservatives. The abovementioned application and protective agents were provided by the manufacturer HBM. RS200 machine (manufactured by MM-Vishay, Malvern, PA, USA) with a cutter of 3.2-mm diameter was used for low-speed drilling. A blind hole with the depth of 5 mm was drilled in ten steps with uniform increments of 0.5 mm. A view of all the holes after drilling on the sluice gate is shown in Figure 10.

The values of the strains ε_a_, ε_b_ and ε_c_ released in the drilling process were recorded with a strain gauge model P3, manufactured by MM-Vishay and subsequently processed and evaluated in accordance with ASTM E 837-13a [27]. Principal normal stresses and their directions in locations RS1 to RS5 were obtained from the values ε_a_, ε_b_ and ε_c_ using the H-Drill software. The values are given in Table 4. Angle *β* indicates the directional deviation of the maximum principal stress from the grid axis *a* (Figure 9), while its positive orientation is considered clockwise. For the stress analysis, values of equivalent stresses were calculated at the drilled points according to von Mises theory

(1) $$\sigma^{von\;Mises}=\sqrt{\sigma_{1}^{2}+\sigma_{2}^{2}+\sigma_{3}^{2}-\left(\sigma_{1}\sigma_{2}+\sigma_{1}\sigma_{3}+\sigma_{2}\sigma_{3}\right)}.$$

The measured values of residual stresses take into account the existing operation (including overload), production technology (welding) and the loss of material due to corrosion. Table 4 shows that, at RS2, RS3 and RS5, the main residual stresses are only tensile stresses. The values of residual stresses did not reach 80% of the material yield strength in any of the measuring points. As a result, these values can be considered valid according to ASTM E837-13a.

## 4. Experimental Determination of Time Changes of Stresses in Selected Places of Sluice Gate during Operation

The aim of the experimental measurement was to take real boundary conditions into account, including bonds in the bearing, load dynamics and other operational impacts. The knowledge of time-dependent stress charts in selected places can be considered, among other things, as a tool for validation of numerical modelling results [31]. Deeper knowledge of these conditions will allow to draw conclusions about stress states in individual modes of operation as well as about residual life of the sluice gate.

**Figure 11:**
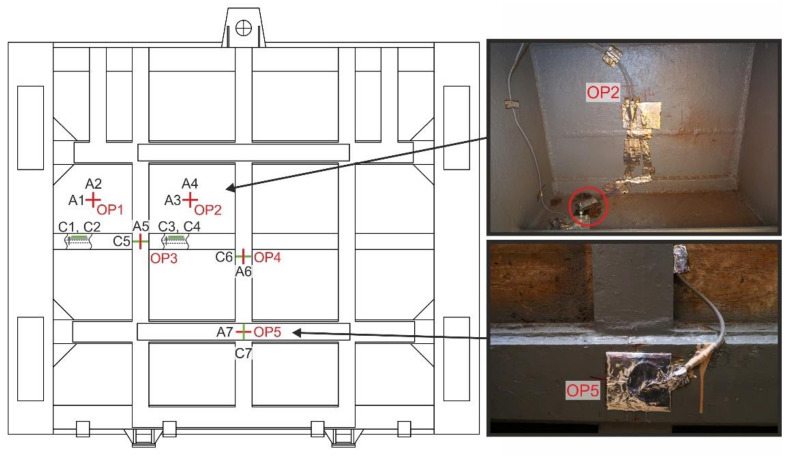
Application points of strain gauges for operational measurement.

Gauge application points were chosen outside local concentrations to allow validation of measured values with results determined by numerical modelling. Such points are flanges of the reinforcing ribs near the axis of symmetry. In order to obtain higher levels of measured strains, the measurement was also performed on the rib in the area with the highest values of hydrostatic pressure. The application points of strain gauges OP1 to OP5 on the sluice gate are shown in Figure 11. Strain gauges type XY91-10/120 manufactured by HBM (Darmstadt, Germany), series A 405/33, serial number 812056981 with ohmic resistance of 120 Ω ± 0.5% were used for the measurement. Deformation sensitivity constant k_a_ was 2.05 ± 1% and k_b_ was 2.01 ± 1%. The effects of temperature on the measured values were compensated by connecting active gauges together with compensation gauges to a half bridge. At measuring points OP1 and OP2, a biaxial (planar) stress was assumed with directions of main stresses identical to horizontal and vertical directions. The axes of active measuring grids A1 to A4 at locations OP1 and OP2 were identified with these directions. Compensation gauges C1 to C4 were applied to unloaded steel sheets which were lightly attached to the sluice gate. Measuring grids of active and compensation gauges at OP1 and OP2 were connected to adjacent branches of the Wheatstone bridge with sensitivity n = 1, thus compensating for the effect of temperature change. At the measuring points OP3, OP4 and OP5, i.e., in flange centres, a uniaxial (linear) stress was assumed with the direction of the main stress identical to the longitudinal direction of the respective flange. The axes of active grids A5 (vertical direction), A6 (vertical direction) and A7 (horizontal direction) were identified with each of these directions (Figure 11). Measuring grids C5, C6 and C7 as parts of strain gauges at OP3 to OP5 fulfilled both active and compensatory roles. Figure 11 shows an example of applied active and compensatory strain gauges after application of protective agents at OP2 and OP5.

**Figure 12:**
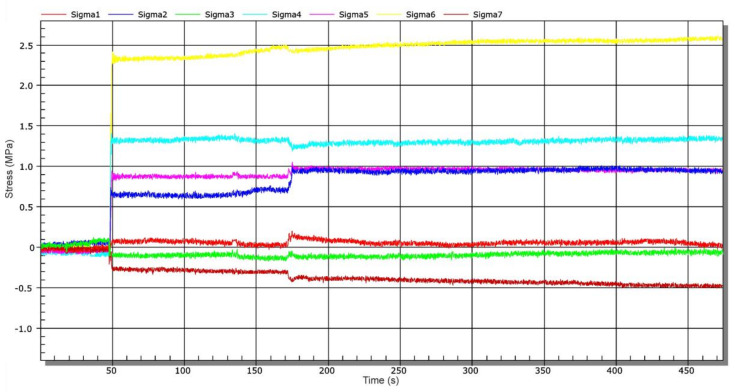
Time-dependent stress chart during lifting of the sluice gate from supporting elements—stage 1.

**Figure 13:**
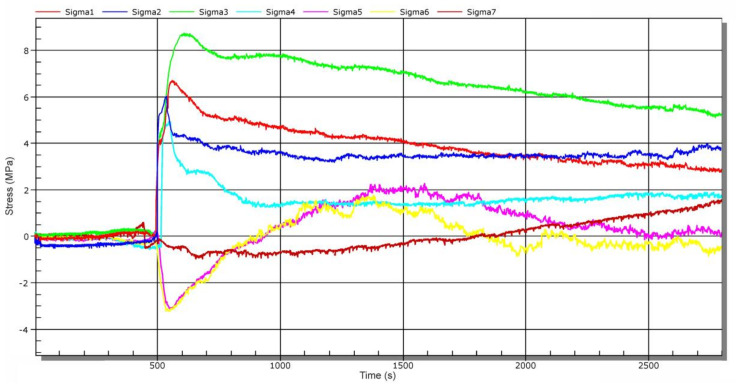
Time-dependent stress chart during sluice gate lowering—stage 2.

Quantum MX840 strain gauge (manufactured by HBM, Darmstadt, Germany) with Catman Easy (version 3.5) evaluation software was used for the operational measurement. Since sluice gate lowering at low speed (approximately 0.7 ms^−1^) was considered, a sampling frequency of 50 Hz was set. The operational measurement was divided into several stages [16]. In the next part, time-dependent recalculated stresses for each stage are presented separately. Prior to each measurement, the strain gauge was balanced, i.e., time increments (changes) of stresses were monitored only for a given stage (not relative to the initial state of the first stage). Figure 12 shows a corresponding time record of stresses when lifting the sluice gate from the rest position (loosely placed on the supporting elements). The values ranged from −0.5 MPa to 2.5 MPa. This is the effect of its own weight. After the sluice gate was lifted to the height of about 1 m, wooden supports were removed and then the sluice gate was lowered to the level of the connecting water tunnel—stage 2. Figure 13 shows a time-dependent chart of stress changes during sluice gate lowering. At a time of about 550 s, there was a more significant change in stress caused by water pressure on the bottom of the sluice gate. Lowering took about 30 min.

**Figure 14:**
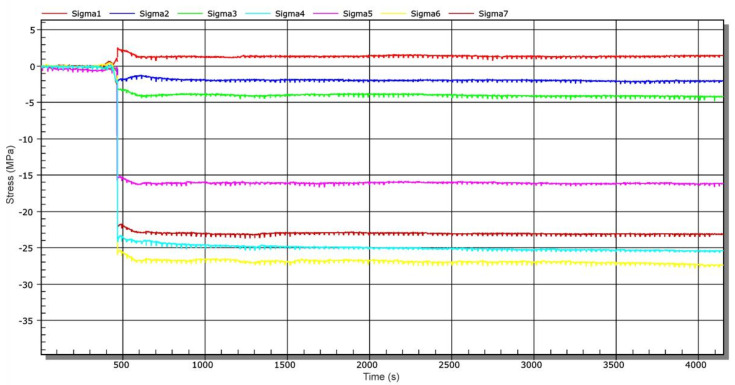
Time-dependent stress chart during water discharge from the water tunnel—stage 3.

After the sluice gate was lowered to the level of the connecting tunnel (Figure 1, position 3), water was discharged from this water tunnel—stage 3. Figure 14 shows a time-dependent chart of stress changes during water discharge from the connecting water tunnel. At a time of about 460 s, a shock was recorded, which was accompanied by a sound effect acting on the measuring station. Most likely, it was the setting of the sluice gate in its final position from the height of approximately 10 cm. The stated value is indicative and was estimated on the basis of vertical movement of the bundle of measuring cables (from strain gauges). At that time, no significant change in stress levels was recorded due to a set sampling frequency of 50 Hz. From a time-dependent chart of stresses, as shown in Figure 14, maximum values of compressive stresses of about 27 MPa were identified in the flanges. After complete drainage of water from the connecting water tunnel (end of stage 3), a 2-week technological shutdown was initiated in connection with tightness check of the sluice gate.

**Figure 15:**
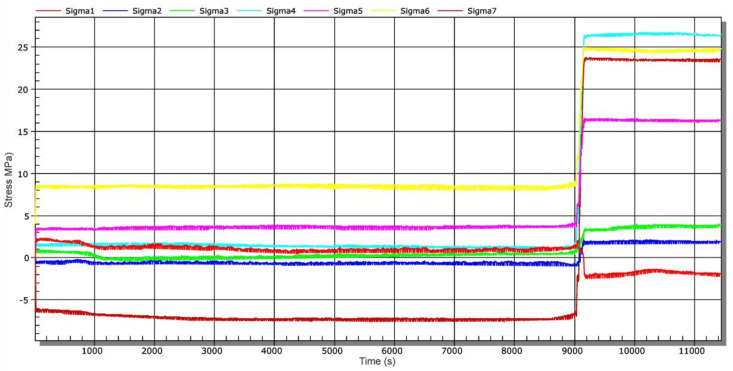
Time-dependent stress chart during water discharge from the water tunnel—stage 3.

After this time, other operational measurements were performed. Figure 15 shows a time-dependent chart of stress changes during water inlet into the connecting water tunnel—stage 4. The water inlet was carried out by gradual lifting of the sluice gate approximately 20 cm above the threshold of the connecting water tunnel (not to position 2, Figure 1). This means that the inlet to the channel has not been completely opened (Figure 1, position 2). The measurement started simultaneously with the sluic gate lifting process. At approx. 9150 s, the connecting water tunnel was completely filled, which is documented by a step stress change (Figure 15).

As part of the control operational measurement, processes of discharge (stage 5) and filling (stage 6) of the connecting water tunnel were carried out once again. Table 5 shows maximum stress increments registered in individual modes of operational measurement. Based on the comparison of measured data for the same operational modes of stages 3 and 5 or stages 4 and 6, a very good agreement was reached. It follows from the above mentioned that strain gauges were fully functional even after a two-week outage.

**Figure 16:**
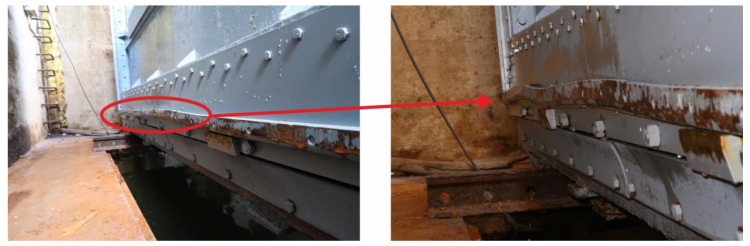
Deformed bottom part of the bronze sealing.

After the blocking sluice gate was lifted above the water level (stage 6), a permanent deformation of the bronze sealing (Figure 16) was found, which probably occurred due to the sudden settlement of the sluice gate during its first lowering (stage 3). However, even such a significant deformation during the monitored period (approximately 2 weeks) did not cause water leakage into the connecting water tunnel. Nevertheless, in order to ensure safe and reliable operation of the sluice gate, it is necessary to repair the seal and to more closely monitor the process of sluice gate lowering.

## 5. Determination of Stress Ratios in Load-Bearing Elements of the Sluice Gate by the Finite Element Method

**Figure 17:**
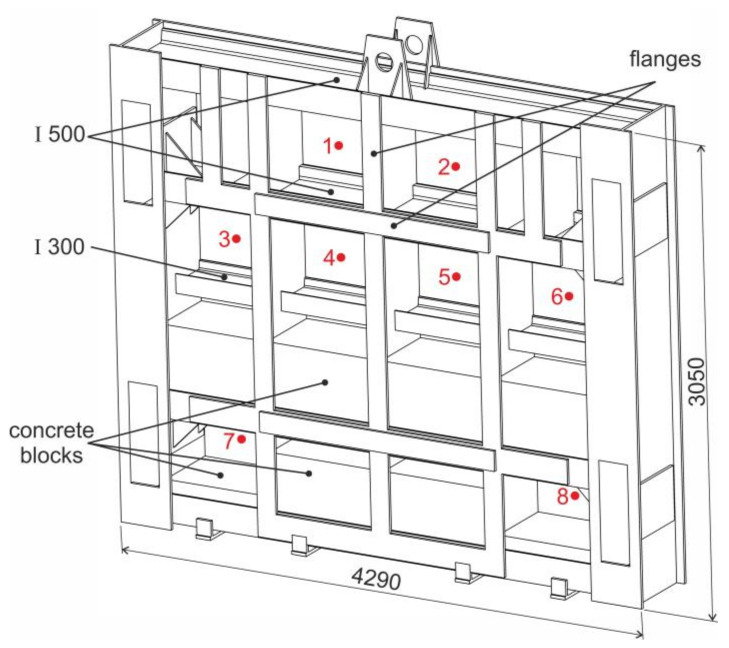
Deformed lower part of the bronze sealing.

For the purposes of numerical modelling as well as for the analysis of deformations and stresses under operating load, it was necessary to determine the shape and basic dimensions of the sluice gate, including the thickness of sheets. In this case, it was a matter of determining the actual thickness, which took into account mainly corrosive losses of the material. The thickness measurement was performed by the measuring system TG 400 (NDT Systems, Huntington Beach, CA, USA) with measuring accuracy 0.01 mm. It is a small, easy-to-use, contact ultrasonic device with high resolution for direct reading of the measured value. Prior to the measurement, calibration was performed on steel reference gauges. When selecting places for thickness measurement of individual elements of the sluice gate, real operating conditions and accessibility of measuring points were taken into account. For this reason, the following measuring points were selected for individual elements of the sluice gate: rear plate—8 points, side elements—8 points, profile I 500—6 points, profile I 300—4 points, reinforcement elements—6 points and flanges—12 points. Figure 17 shows an indication of positions for the thickness measurement on the back plate of the sluice gate.

Prior to measurement, each measuring point was gently ground and cleaned of impurities that could affect final results. Using a probe, the thickness of individual elements of the sluice gate (flanges, webs, reinforcing elements and back plate thickness) were measured. At each point, 3 thickness measurements were performed, from which average values were determined. Finally, average thickness values of investigated elements were determined, which are listed in Table 6.

**Figure 18:**
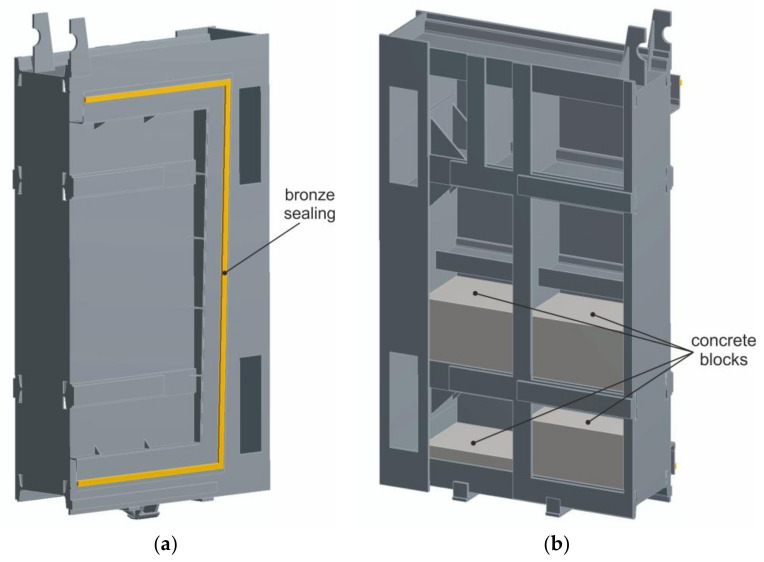
Model of the sluice gate: (**a**) from the side of the connecting water tunnel and (**b**) from the side of the water reservoir.

Obtained dimensions of the sluice gate were subsequently used in the creation of a model for deformation and stress analysis by numerical methods. The finite element analysis was performed for the worst-case scenario, i.e., for dimensions of load-bearing parts rounded down to one decimal place. In order to assess safe operation of the sluice gate, strength and deformation analysis was performed using the finite element method. Since the analysed sluice gate is a symmetrical structure, it was sufficient to solve only half of the model and to use the appropriate boundary conditions in the plane of symmetry of the structure. The created model in the software SolidWorks 2019 is shown in Figure 18. Guide wheels are not part of the model because they serve only for vertical guidance of the sluice gate and are not essential with respect to the performed analysis.

The model is meshed by volume finite elements. The model consists of 297,126 nodes and 145,645 finite elements. The symmetric condition is applied to the half model. The displacement of bronze bead in the normal direction is fixed, and the bottom bumper is supported. In addition to hydrostatic load from water pressure on the side of the water tank (0.215 MPa), the structure is also loaded by own-weight forces taking into account water uplift force (91.4 kN). Since these are constant loads and boundary conditions do not change during the analysis, the calculation is performed using static analysis. During calculation, the parameters of individual materials were considered, which are listed in Table 7.

**Figure 19:**
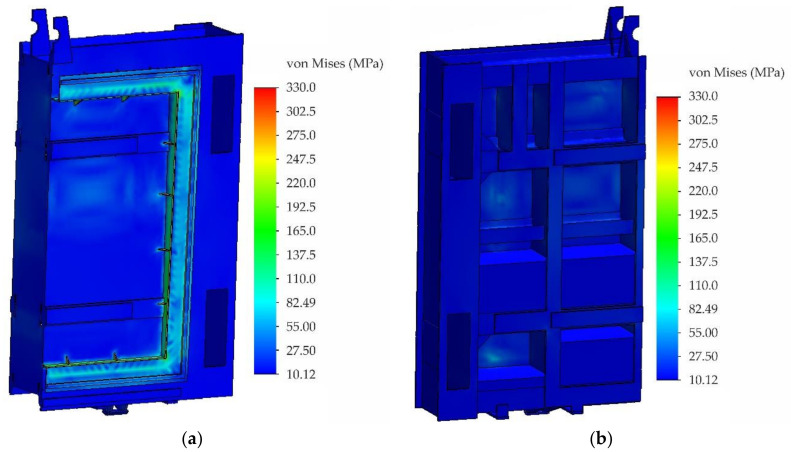
Fields of equivalent stresses according to von Mises theory: (**a**) front view and (**b**) rear view.

This section presents the results of the calculation for the sluice gate at the maximum operating water level 786.10 MAMSL taking into account the nonlinear behaviour of the material above the yield point. During the nonlinear analysis, an elastic-plastic material model with linear reinforcement with yield strength Re = 330 MPa (value determined from tensile test) and with tangent modulus of elasticity E_t_ = 11,000 MPa was applied. The field of equivalent stresses according to von Mises theory is shown in Figure 19. For better identification of critical points, the maximum stress was limited to 330 MPa when plotting. From Figure 19, it is clear that the highest levels of equivalent stresses are at locations of triangular reinforcements located around the circumference of the steel frame to which the bronze sealing is attached. Maximum values of equivalent stresses around triangular reinforcements reached the value of 360 MPa, thus exceeding the yield strength of the analysed samples. Although values at these critical points exceeded the yield strength of the used material taken from the sluice gate, permanent deformations were not detected during visual inspection.

**Figure 20:**
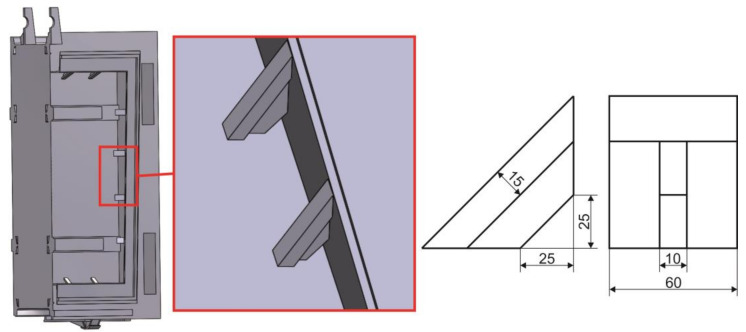
Newly designed reinforcing elements welded to the sealing part of the sluice gate with its basic dimensions.

The purpose of triangular reinforcements is to attach a steel frame used to fasten the bronze seal. Despite the fact that permanent deformations of the triangular reinforcements were not found during visual inspection, there is a realistic presumption of permanent local deformation, which can be considered unsatisfactory from the point of view of safe operation. For this reason, reinforcement by welding a 15-mm thick flange was proposed to remove the identified stress peaks. The newly designed reinforcing elements located in place of the original reinforcing elements are shown in Figure 20. The dimensions of modified reinforcing elements are clearly shown in Figure 20 (right). Only basic dimensions are shown in this figure because the height of the sealing element varies around the circumference and the reinforcement should reach its edge as is the case at present. An important part of this design is an opening in the shape of a right triangle with length of legs 25 mm which is created after welding. The purpose of this hole is to reduce the stress level at the point of its concentration.

**Figure 21:**
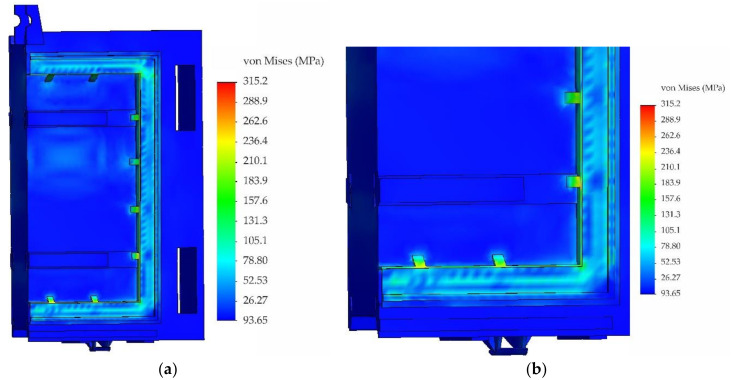
Fields of equivalent stresses according to von Mises theory: (**a**) general view and (**b**) detail.

The modified model was again subjected to nonlinear stress analysis with the same boundary conditions. The field of equivalent stresses on the sluice gate with modified reinforcing elements according to von Mises theory is shown in Figure 21.

The value of maximum equivalent stress in the vicinity of modified reinforcing elements dropped to 315 MPa, i.e., about 15%. The performed numerical calculation of the sluice gate load did not take into account dynamic effects when lowering the sluice gate, the settlement of the bumper zone on the threshold of the tunnel as well as the pressing of the bronze sealing on the inclined surface of the tunnel.

It should be noted that, when assessing the safe and reliable operation of welded structures, it is necessary to consider the manufacturing and assembly inaccuracy, residual stresses from production, etc., which often cannot be precisely defined in boundary conditions of numerical modelling. In this case, the results obtained by numerical modelling were validated with experimental measurements in real operation. Table 8 contains the comparison of stress values determined in directions of active grids of strain gauges under operating load with values determined by numerical modelling.

From the measured time changes of stresses, it is clear that stress values show a very good compliance with values determined by numerical modelling. The differences between numerical and experimental modelling can be explained as follows:

- The numerical calculation takes into account the most unfavourable stress.
- In the computational model, passive relationships between the threshold and the sluice gate were not considered.
- In the experimental verification, the boundary conditions take into account flexibility of the boundaries as well as all local real contact pressures, which is not possible in numerical modelling. Ideal stiff bonds are considered in numerical modelling in contrast to experimental measurements on a real object.

Table 9 lists the values of equivalent stresses which were determined experimentally (residual stresses) for RS1 to RS5 and by numerical modelling for the case of maximum operating load (water discharge from the connecting water tunnel—stage 3). From Table 9, it is clear that the values of superimposed equivalent stresses at the analysed places do not exceed the yield strength of the material and can be considered satisfactory. The table compares the values at the points of the measured residual stresses. The aim was to show that in these places are a higher level of residual stresses and that the values of stresses from the operating load are low in these places. However, the stress values for the maximum operating load were not experimentally measured at these locations but were determined by numerical modelling. Validation of the results of numerical modelling with experimental measurements was performed at OP1 to OP5.

## 6. Conclusions

The paper presents the procedure for assessing safe and reliable operation of the hydroelectric power plant inlet sluice gate operated for more than 60 years. The reason was, among other things, the finding of permanent damage to some parts of the sluice gate (Figure 1). In order to draw conclusions, it was necessary to determine mechanical properties of the used material as well as the loss of material due to corrosion (real dimensions of the structure). The proposed methodology was based on validation of the results of numerical and experimental modelling. The conclusions can be summarized as follows:

- On a simplified model of the sluice gate, the finite element analysis was performed with boundary conditions corresponding to the most unfavourable scenario, i.e., operation at the highest water level in the water reservoir. Numerical modelling identified critical points located in the area of reinforcing elements where the levels of equivalent stresses reached almost 360 MPa. Values at these critical points exceeded the yield strength of the used material used, but permanent deformations were not detected during visual inspection. Nevertheless, a structural modification of these critical points was proposed, which reduced the stress values by about 15%.
- The results of numerical modelling were verified at selected locations of the analysed sluice gate by operational experimental measurements using strain gauges. The maximum values of normal stresses in the assessed places reached about 27 MPa. The advantage of operational measurement is identification of the real behaviour of the structure. In this case, the registered shock was probably caused by an imperfect settlement of the sluice gate in the desired position. Since this shock was not expected during the operational measurement, the measurement was performed with a sampling frequency of only 50 Hz. At this frequency value, it was not possible to evaluate maximum stress peaks measured at the analysed locations. Numerical modelling by the finite element method did not analyse this state, as the exact definition of an unexpected (unknown) load in boundary conditions is practically impossible. Among other things, this is a condition that should not occur if handled correctly.
- When assessing safe operation, the levels of experimentally measured residual stresses were also considered, which in some places reached up to 190 MPa. Although the levels of resulting (superimposed) stresses are higher than is usually allowed in such structures, it can be stated that, in the current state of structure, the sluice gate can be operated safely.

Based on the analysis of numerical and experimental modelling, the following recommendations were made:

- Carry out a structural modification of the reinforcing elements (triangles).
- Gradual step lowering of the sluice gate, which should be realised in the last quarter of the closure with a step downwards by approx. 100 mm and subsequent slight lift to eliminate any jamming of the sluice gate. If this principle is fulfilled, the operation of the sluice gate can be considered safe.
- When manufacturing a new sluice gate, use a material with minimum strength of 500 MPa and remove residual stresses after.

## Figures and Tables

**Table 1:** Mechanical properties of the material.

Sample	R_p0,2_ (MPa)	R_m_ (MPa)
1	332	491
2	328	486

**Table 2:** Chemical composition of the analysed material (%).

Nr.	Fe	C	Si	Mn	P	S	Cu	Al	Cr	Mo	Ni	V	Ti	Nb	Co
1.	98.98	0.127	0.201	0.491	<0.002	<0.002	0.137	<0.002	0.020	0.005	0.024	<0.002	<0.002	<0.002	<0.002
2.	98.97	0.121	0.211	0.494	<0.002	0.003	0.136	<0.002	0.022	0.007	0.022	<0.002	<0.002	<0.002	0.009
3.	98.93	0.158	0.209	0.479	<0.002	<0.002	0.137	<0.002	0.021	0.020	0.026	<0.002	<0.002	0.004	0.013
Mean value	98.96	0.135	0.207	0.488	<0.002	<0.002	0.137	<0.002	0.021	0.011	0.024	<0.002	<0.002	0.003	0.007

**Table 3:** Brinell hardness and impact toughness.

Sample	HBW	KC (J)	KCV (J⋅cm^−2^)
1	132; 134; 137	72	90
2	132; 135; 137	77	96

**Table 4:** Values and directions of residual stresses according to ASTM E 837-13a.

Location	σ_max_ (MPa)	σ_min_ (MPa)	Angle *β* (°)	σ_red_ (MPa)
RS1	119	−67	−75	163.17
RS2	184	65	0	161.62
RS3	185	141	32	167.39
RS4	54	−126	−78	159.99
RS5	134	84	42	117.29

**Table 5:** Maximum values of normal stresses in the direction of active gauges.

Operational Measurement	σ_max_ (MPa) for Location						
	OP1/A1	OP1/A2	OP2/A3	OP2/A4	OP3/A5	OP4/A6	OP5/A7
stage 1	0.1	1.0	−0.1	1.3	1.0	2.5	−0.5
stage 2	6.6	6.0	8.7	4.9	−3.1	−3.1	−0.9
stage 3	1.5	−2.2	−4.1	−25.0	−16.2	−27.2	−23.0
stage 4	−2.1	1.9	3.9	26.5	16.2	24.8	23.8
stage 5	−1.1	−1.6	−4.9	−26.2	−18.1	−24.5	−20.5
stage 6	−0.5	1.1	4.1	26.3	18.7	27.3	19.3

**Table 6:** Average thickness values of the load-bearing elements of the sluice gate.

Evaluated Element	Number of Measuring Points	Average Thickness (mm)	Thickness Applied in the FEM Model (mm)
rear plate	8	19.75	19.70
side elements	8	20.09	20.00
profile I 500	6	18.01	18.00
profile I 300	4	10.82	10.80
reinforcing elements	6	15.35	15.30
flanges	12	19.27	19.20

**Table 7:** Material properties of used materials.

Element	Young’s Modulus E (MPa)	Poisson’s Ratio μ (¬–)	Density ρ (kg⋅m^−3^)
welded element (sluice gate)	2.06⋅10^5^	0.3	7.900
concrete	3⋅10^5^	0.2	2.300
bronze sealing	1.11⋅10^5^	0.34	8.800

**Table 8:** Comparison of normal stress values in the direction of active gauges.

Type of Analysis	σ_max_ (MPa) for Location						
	OP1/A1	OP1/A2	OP2/A3	OP2/A4	OP3/A5	OP4/A6	OP5/A7
FEM	−4.2	−1.1	−14.7	−29.7	−24.4	−33.2	−20.8
operational measurement—stage 3	1.5	−2.2	−4.1	−25.0	−16.2	−27.2	−23.0
operational measurement—stage 5	−1.1	−1.6	−4.9	−26.2	−18.1	−24.5	−20.5

**Table 9:** Equivalent stress values at drilling points.

Type of Analysis	σ_red_ (MPa) for Location				
	RS1	RS2	RS3	RS4	RS5
Hole drilling method	163.17	161.62	167.39	159.99	117.29
FEM	7.8	4.4	31.5	3.1	6.3
